# Photoperiod Regulates Lean Mass Accretion, but Not Adiposity, in Growing F344 Rats Fed a High Fat Diet

**DOI:** 10.1371/journal.pone.0119763

**Published:** 2015-03-19

**Authors:** Alexander W. Ross, Laura Russell, Gisela Helfer, Lynn M. Thomson, Matthew J. Dalby, Peter J. Morgan

**Affiliations:** Rowett Institute of Nutrition and Health, University of Aberdeen, Aberdeen, Scotland, United Kingdom; Hosptial Infantil Universitario Niño Jesús, CIBEROBN, SPAIN

## Abstract

In this study the effects of photoperiod and diet, and their interaction, were examined for their effects on growth and body composition in juvenile F344 rats over a 4-week period. On long (16L:8D), relative to short (8L:16D), photoperiod food intake and growth rate were increased, but percentage adiposity remained constant (ca 3-4%). On a high fat diet (HFD), containing 22.8% fat (45% energy as fat), food intake was reduced, but energy intake increased on both photoperiods. This led to a small increase in adiposity (up to 10%) without overt change in body weight. These changes were also reflected in plasma leptin and lipid levels. Importantly while both lean and adipose tissue were strongly regulated by photoperiod on a chow diet, this regulation was lost for adipose, but not lean tissue, on HFD. This implies that a primary effect of photoperiod is the regulation of growth and lean mass accretion. Consistent with this both hypothalamic *GHRH* gene expression and serum IGF-1 levels were photoperiod dependent. As for other animals and humans, there was evidence of central hyposomatotropism in response to obesity, as *GHRH* gene expression was suppressed by the HFD. Gene expression of hypothalamic *AgRP* and *CRH*, but not *NPY* nor *POMC*, accorded with the energy balance status on long and short photoperiod. However, there was a general dissociation between plasma leptin levels and expression of these hypothalamic energy balance genes. Similarly there was no interaction between the HFD and photoperiod at the level of the genes involved in thyroid hormone metabolism (*Dio2*, *Dio3*, *TSHβ* or *NMU*), which are important mediators of the photoperiodic response. These data suggest that photoperiod and HFD influence body weight and body composition through independent mechanisms but in each case the role of the hypothalamic energy balance genes is not predictable based on their known function.

## Introduction

For many mammals physiological indices such as food intake, growth, energy balance and reproductive status are not fixed, but vary with the season of the year [1]. The evolutionary advantage of these changes is to ensure that animals are optimally adapted for survival to their changing seasonal environment. The changes in physiology are often marked in magnitude, tightly controlled by photoperiod and homeostatic. They are therefore unlike changes induced by environmental stresses such as food deprivation or overnutrition [2]. Evidence to date also suggests that the marked changes in seasonal energy balance and growth involve novel neuroendocrine mechanisms and on this basis may provide an opportunity to develop new insights into the neuroendocrine control of physiology.

Studies on a number of species have indicated that the effects of photoperiod on the neuroendocrine system are mediated through the hormone, melatonin, which acts on the pituitary, pars tuberalis [3]. In turn this triggers downstream alteration of thyroid hormone and retinoic acid metabolism within the tanycyctes and ependymal cells around the third ventricle of the hypothalamus [3,4]. It is thought that changes in both thyroid hormone and retinoic acid bioavailability within the hypothalamus alter the neuroendocrine systems controlling growth, energy balance and reproduction [5,6].

The juvenile F344 rat has become a particularly useful model for the study of the effects of photoperiod on growth, body weight and energy balance [7]. Under short photoperiod food intake is reduced, growth rates are slower, and body weights lower relative to rats held under long photoperiod. The slower rate of growth under short photoperiod is associated with lower hypothalamic *GHRH* gene expression and lower serum IGF-1 levels [7]. Consistent with the reduced levels of food intake under short photoperiod are lower levels of the orexigenic gene, *AgRP*, in the arcuate nucleus (ARC). Paradoxically, despite lower food intake, expression of the orexigenic gene *NPY* is higher in the ARC in short relative to long photoperiod. It has been suggested that the main role of NPY under these conditions may be to inhibit GHRH and hence the growth axis, leaving AgRP as the main regulator of food intake [7].

In both animal models and humans it has been shown that the neuroendocrine growth axis is influenced by either obesity status or feeding on a high fat diet, leading to increased adiposity. In humans the amplitudes of both spontaneous and stimulated pulsatile GH secretion are blunted [8,9,10]. Likewise, in rats and mice, obesity is associated with hyposomatotropism [11,12,13,10,14,15,16]. The origins of this reduced somatotrophic activity appears to vary according to the model examined. For example in Zucker rats, a genetic model of obesity, both *GHRH* and *SRIF* gene expression are suppressed in obese relative to lean rats, indicating hypothalamic involvement in the hyposomatotropism [14,13]. By contrast in ob/ob and db/db mice as well as diet-induced obese mice and rats, there is no central effect suggesting a more peripheral origin to the condition [17,15,16,18]. In humans, childhood obesity is associated with increased height (growth) as well as adiposity [19]. At the same time, in both children and juvenile rodent models, obesity is associated with suppressed activity of the growth hormone axis [19,10]. Thus there may not always be a direct correlation between growth hormone secretion, IGF-1 levels and growth.

The susceptibility of photoperiodic species to high fat diets and adiposity seems to vary according to species. Siberian hamsters, Shaw’s Jird and Bank voles have each been shown to be resistant to diet-induced obesity when fed a HFD [20,21,22]; by contrast Syrian hamsters become obese [23]. It has been suggested that those animals which lose body or fat mass in response to short photoperiod as part of their normal seasonal strategy are resistant to diet induced obesity, whereas those that gain weight or increase fat mass in response to short photoperiod are prone to obesity on a HFD [20].

Juvenile F344 rats gain less weight on short photoperiod than on long photoperiod [24,7], and so might be predicted to be resistant to diet-induced obesity. A recent study of F344 rats, which were fed a very high fat diet, supports this prediction, based on epididymal fat and leptin levels [25]. However in this study, a more comprehensive assessment of body composition has been used and we show that photoperiodically-sensitive F344 rats are susceptible to diet-induced obesity. Moreover in the light of the suppressive effects that diet induced obesity and increased adiposity have on the somatotrophic axis in both juvenile and adult rodents models and humans, we sought to examine the potential interactions between the effects of consuming a HFD and photoperiod on the regulation of growth, food intake and body weight in juvenile F344 rats.

## Materials and Methods

### Animals

Male F344/NHsd rats aged 3–5 weeks were obtained from Harlan, USA. Initially the 40 rats were group housed, 5 per cage for one week under 12 h light:12 h dark photoperiod with *ad libitum* access to water and standard chow (CRM(P), Rat and Mouse Breeder and Grower, standard AIN93G-based pelleted diet, product code 801722, Special Diet Services, Witham, Essex, UK). This diet provided 3.59 kcal/g of energy with 9.08% calories as fat, 68.9% as carbohydrate and 22.0% as protein, and contained fat, carbohydrate and protein at 3.4, 4.2 and 18.4%w/w respectively. Rats (weight range of 77.7–106.4g, median 86.6g), were then randomly assigned to four weight-matched groups (n = 10 per group) and housed singly in standard rat cages type RC2/f, size 39.5 x 57.0 x 22.5cm, w x l x h. Two groups were switched to a short photoperiod (8 h light: 16 h dark; SD) and the remaining two groups to a long photoperiod (16 h light: 8 h dark; LD). All rats had continuous *ad libitum* access to water. One group in each photoperiod was provided *ad libitum* either a high fat diet, or the standard chow diet, for 4 weeks. The high fat diet (TD.06415) was obtained from Harlan Teklad Custom Diets (Madison, USA) and provided 4.6 kcal/g with 44.8% of calories in the form of fat, 36.2% as carbohydrate and 19.0% as protein and contained fat, carbohydrate and protein at 22.8, 41.4 and 21.7%w/w respectively.

This experiment was repeated to provide sufficient tissues and brains for analysis (repeat study weight range of 124.6–150.0g, median 141g). Group sizes were initially selected based on previous experience where F344 rats showed a significant weight difference after 4 weeks in photoperiod, then in the repeat study, these comprised of 8 rats per group, determined by a power calculation based on results obtained. For example, for gene expression changes that typically showed a difference in expression of ~60% with a standard deviation of 29.6, an R value of 1.99 (where R is the ratio of the difference required to detect the between-animal standard deviation) then, in a power calculation for 95% confidence, 8 animals per group were required. Apart from room lighting changes, all other environmental conditions were the same; temperature was 21°C +/- 2°C, relative humidity of 55% +/- 10% and average light intensity of 150 lux, with plastic tunnel and shredded paper enrichment and G6 woodchip bedding. Daily visual health checks on all rats were performed and no welfare-related interventions were required.

Body weights and food intakes were recorded and rats were scanned by magnetic resonance imaging (MRI) for repeated fat and lean tissue measurements on a weekly basis throughout the study. The MRI scanner (EchoMRI, Houston, Texas, USA) was calibrated as described by Lobley and colleagues [26]. Animals were scanned to give triplicate readings for fat mass within 1g.

Animals were assigned random numbers before killing which allowed all analyses to be conducted blind to the groupings. Animals were killed by terminal anaesthetic using isoflurane, followed by decapitation at approximately 3 hours after lights on (ZT3). Trunk blood was collected for serum and plasma and brains were immediately removed and frozen on dry ice, while livers, kidneys, muscle, epididymal fat pads and pituitaries were collected and wet weights recorded. ZT3 was chosen as a single time point to assess the effects of photoperiod as we have previously shown that the dark/light transition synchronises the expression of the clock gene Per1 and the transcription factor ICER [27]. On this basis it is used as a standard time point to determine the effect of photoperiod on hypothalamic gene expression [5,7,28]. It is also important to note that previous studies have shown the validity of using single time-point determinations to assess the effect of photoperiod, since there is minimal diurnal variation in the hypothalamic genes or hormone levels studied in this paper [29].

### Ethics statement

All animal procedures were performed according to the Animals (Scientific Procedures) Act, 1986, were licenced by the UK home office under Project Licence PPL60/3615 and PPL60/4282 and approved by the local ethics committee at the University of Aberdeen, Rowett Institute of Nutrition and Health (Approval numbers SA06/17E and SA12/10E).

### Gene Expression Studies

Coronal sections (20 μm) of rat forebrains were cut from -4.36 mm to -2.16 mm relative to Bregma for the ARC and -2.16 mm to -0.96 mm for the paraventricular nucleus (PVN) according to the rat atlas [30] and collected onto poly-lysine coated glass slides as described previously [7]. Riboprobes for in situ hybridization were prepared from cDNA templates that were cloned as described previously for *NPY* [31], *NMU* [32], *pro-opiomelanocortin (POMC)* and *Agouti-related peptide (AgRP)* [33], *corticotrophin releasing hormone (CRH)* [34], *types 2 and 3 deiodinases (Dio2 and Dio3)* [35] and *growth hormone releasing hormone (GHRH)* [7]. cDNA fragment of the rat *thyroid stimulating hormone β subunit (TSHβ)* (505 bp), was amplified by PCR using standard conditions from F344 rat hypothalamic cDNA, using the forward primer 5’-CCGAAGGGTATAAAATGAACAGAG and reverse primer 5’-ACCAGATTGCATTGCCATTACAGT designed against the rat *TSHβ* cDNA sequence (GenBank accession number XM_008761373). The amplified product was cloned into pCR-Blunt 4-TOPO vector (Invitrogen, Paisley, UK) following the manufacturer’s protocol. The sequence was verified by sequencing with Beckman Coulter sequencing chemistry on a CEQ8000 automated sequencer (Beckman Coulter UK Ltd, High Wycombe, Bucks, UK). The TSH*β* template was linearised using *Not*1 and *Spe*1 restriction enzymes for use with T3 and T7 polymerases to generate antisense and sense riboprobes respectively.

Messenger RNA levels were measured by in situ hybridization (ISH) as previously described in detail [33]. Briefly, brain sections were fixed, acetylated (with the exception of NPY) and hybridized overnight at 58°C with sense and antisense riboprobes labelled with ^35^S (1.0–1.5 x 10^10^dpm/L). Slides were treated with RNase A, washed at 60°C in 0.1x sodium citrate salt for 30 minutes, dried and then apposed to Hyperfilm Max autoradiographic film (Amersham Pharmacia Biotech UK Ltd., Buckinghamshire, UK). A standard curve was generated with ^14^C microscales (Amersham Pharmacia Biotech UK Ltd., Buckinghamshire, UK) and used to calculate gene expression levels (integrated optical density) using ImagePro Plus, version 6 (Media Cybernetics, Buckinghamshire, UK). No signals were detected for any of the sense probes in the hypothalamus.

### Circulating Hormones and Metabolites

All serum samples were measured in duplicate with leptin levels using a Linco Research rat leptin radioimmunoassay kit (Millipore (UK) Limited, Watford, UK), while serum insulin-like growth factor-1 (IGF-1) levels were measured using an Octeia immunoenzymometric assay kit (IDS Ltd., Boldon, UK) suitable for use with rat/mouse samples. Growth hormone levels were measured using a rat/mouse growth hormone ELISA kit (Millipore (UK) Limited, Watford, UK) in pituitary extracts prepared according to the manufacturer’s protocol.

Plasma glucose, non-esterified fatty acid (NEFA) and triglyceride levels were determined using a Konelab 30 machine (Thermo Fisher Scientific, Basingstoke, UK). The sensitivity of the assays were 0.34 mmol/L, 0.04 mmol/L and 0.06 mmol/L and the intra assay CVs 0.35%, 2.0% and 2.9%, respectively.

### Statistics

Body weight, food intake and body composition data were analysed via a repeated measures three-way analysis of variance on the rat groups (Genstat version 10, Rothamsted Research, UK). Tissues, gene expression and hormone data were analysed by a two-way analysis of variance with use of the Holm-Sidak post-test, followed by one-way analysis of variance using multiple comparison tests with either Fisher LSD method or Dunn’s method on ranks and t-test where appropriate on treatment groups (Sigma Plot 12, Systat Software Inc., London, UK). Means without a common letter were statistically different with statistical significance set at p<0.05.

## Results

### Photoperiod and high fat diet on food intake, body weight and body composition

**Fig 1:**
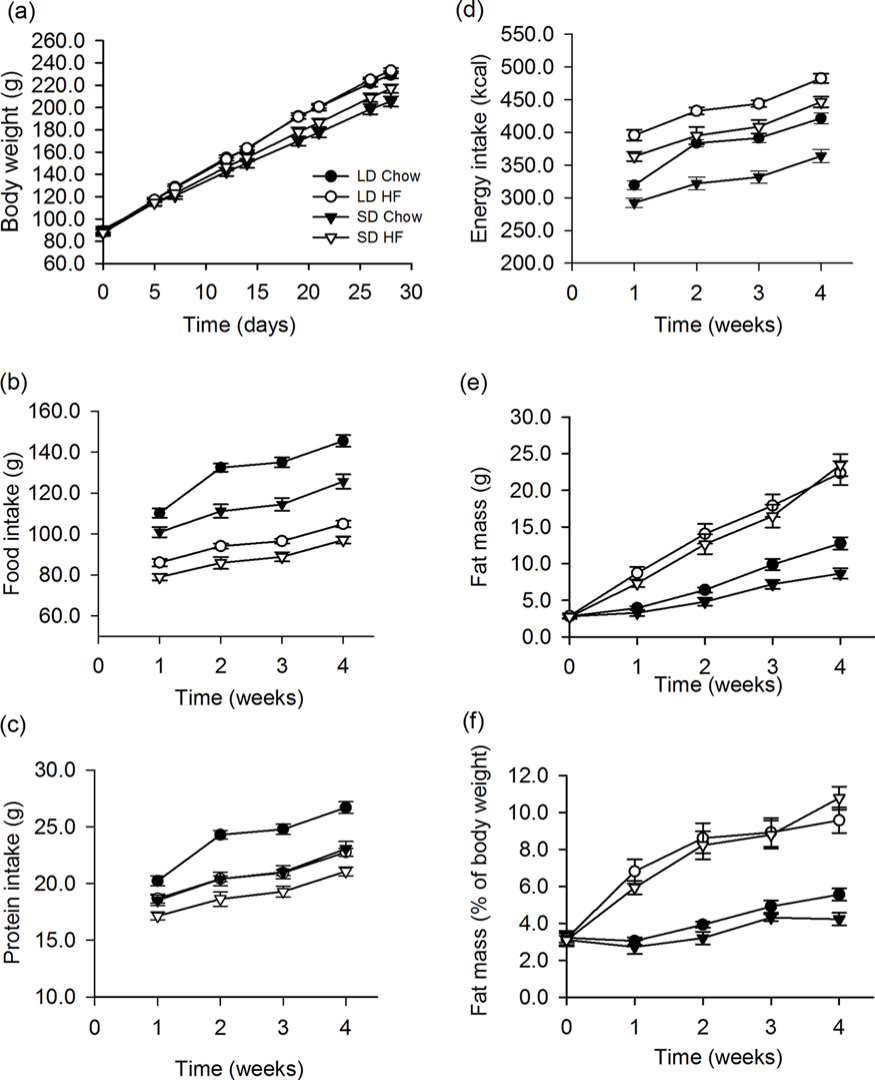
Effect of photoperiod and high fat diet (HFD) on body weight, food, protein and energy intake and body fatness of juvenile F344 rats over 4 weeks of treatment. (a) Body weight was significantly higher in LD compared to SD (p<0.001) on chow diet with no effect of a HFD after 4 weeks. (b) Food intake in grams was significantly higher in LD than SD on chow diet (p<0.001) and these intakes were higher than the intakes of the HFD fed rats (p<0.001). There was a small photoperiodic difference in food intake when fed the HFD (p = 0.03). (c) Protein intake was higher in the LD than the SD chow fed rats (p<0.001). HFD fed rats showed lower protein intakes than the chow fed rats (LD; p<0.001, SD; p = 0.008) and there was a significant effect of photoperiod (p = 0.015). (d) Energy intakes were higher in LD than SD rats fed either chow (p<0.001) or HFD (p = 0.004), while rats fed the HFD had higher energy intakes than chow in both photoperiods (p<0.001). (e) Fat mass measured by Echo MRI was slightly higher in LD than SD rats on chow (p = 0.026), while the HFD fed rats did not differ in their intakes with photoperiod. The HFD fed rats had markedly higher fat masses that were significantly higher than chow fed rats (p<0.001). (f) A HFD markedly increased adiposity as a percentage of body weight compared to chow fed rats and to a similar level in rats on both LD and SD (p<0.001) with no effect of photoperiod. In all figures HF refers to HFD fed rats.

Juvenile F344 rats reared from 4–6 weeks of age on either long or short photoperiod showed divergent body weight. Those on short photoperiod were 14% lower (P<0.001) in weight after 4 weeks relative to those on long photoperiod. On a HFD there was no significant additional effect on body weight in rats reared on either LD or SD (Fig. 1a). In the SD HFD fed group, one rat failed to respond to photoperiod and gained weight similar to the LD rats. In addition, a rat from the SD chow fed group failed to thrive, gaining less weight than the remainder of the group. These two rats were considered outliers and were omitted from all analyses, thus the SD chow and SD HFD groups each had n = 9 rats in the analyses.

Consistent with the difference in body weights on LD and SD, there was a clear LD-SD difference in both food and protein intakes (both p<0.001) when fed a chow diet (Fig. 1b,c). However, when fed a HFD diet, food and protein intakes were lower than the food and protein intakes on a chow diet, for both LD (food and protein, p<0.001) and SD rats (food, p<0.001, protein p = 0.008) (Fig. 1b,c). In addition there were small differences in food (P = 0.03) and protein (p = 0.015) intakes between LD and SD rats on the HFD (Fig. 1b,c). Energy intake was higher in LD than in SD when fed either chow (p<0.001) or HFD (p = 0.004). In addition, energy intake on a HFD was significantly higher than chow in both photoperiods (p<0.001), (Fig. 1d). This is reflected in changes in body composition where there was a small but significant effect of photoperiod on fat mass between rats fed on chow in LD and SD, (Fig. 1e). Consumption of a HFD markedly increased adiposity to a similar level in terms of gross fat (p<0.001) and fat as a percentage of body weight (p<0.001) in rats on both LD and SD within 1 week (Fig. 1e,f). This effect of HFD was maintained over 4 weeks, but there was no significant effect of photoperiod (Fig. 1e,f).

**Fig 2:**
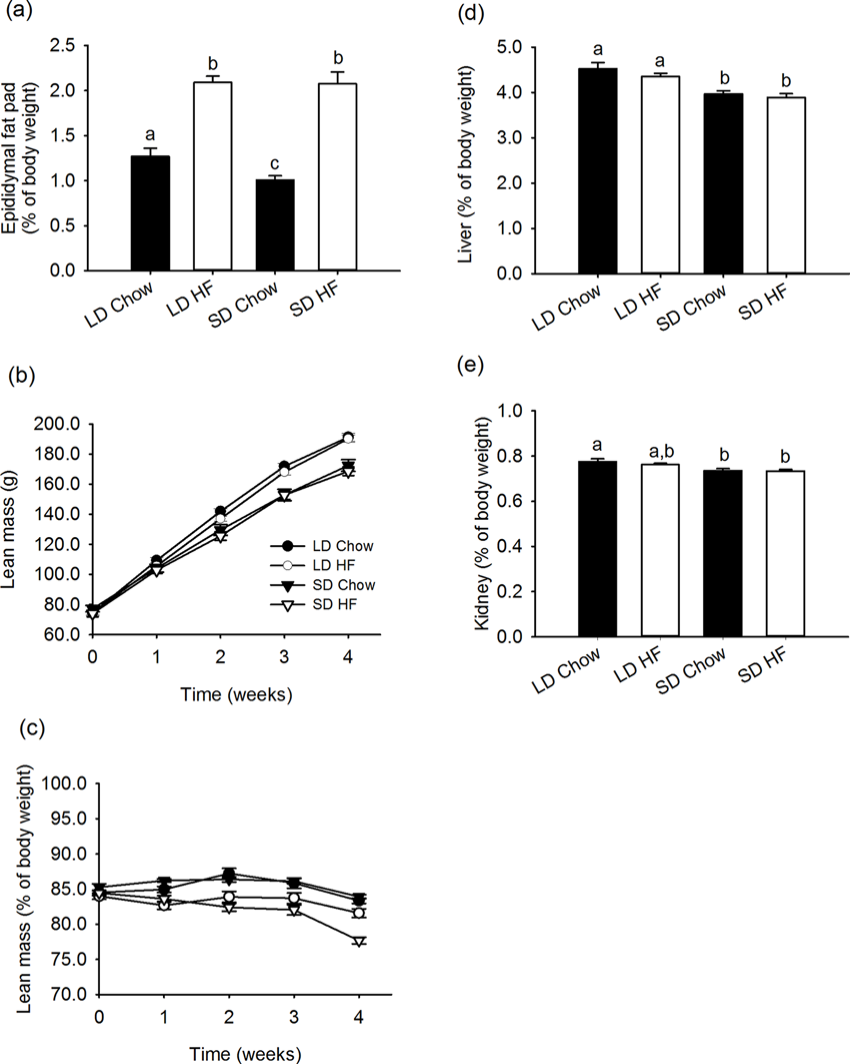
Effect of photoperiod and high fat diet (HFD) on epididymal fat, lean mass and liver and kidney weight of juvenile F344 rats over, or after, 4 weeks of treatment. (a) Epididymal fat pad mass as a percentage of body weight, was greater in LD than SD chow fed rats (p = 0.048). The fat pads masses were significantly greater with HFD feeding than chow in both photoperiods (p<0.001), but there was no effect of photoperiod with HFD feeding. (b) Absolute lean mass was higher in LD than SD rats fed either chow or HFD. The lean masses were not changed by HFD feeding. (c) Lean mass as a percentage of body weight was not significantly different between LD and SD chow fed rats. The lean masses of HFD fed rats were lower than those of the chow fed rats with the SD HFD fed rats being significantly leaner than the LD HFD fed rats (p<0.001). (d) Liver mass was lower in SD than LD chow and HFD fed rats (both p<0.05). The HFD did not impact on the liver masses in either photoperiod. (e) Kidney mass was also lower in both SD chow and HFD fed rats than chow fed LD (P<0.05).

Further effects of photoperiod and HFD diets on body composition are shown in Fig. 2. The changes seen in fat mass seen by MRI are similarly reflected by changes in epididymal fat mass in terms of both photoperiod with chow (p = 0.048) and significant increases with HFD feeding (p<0.001), (Fig. 2a). A clear effect of photoperiod on absolute lean (fat free) mass was observed, being significantly higher in chow and HFD fed LD than SD rats (p<0.001), yet there was no significant effect of HFD on lean mass under either photoperiod (Fig. 2b). In terms of lean mass as a percentage of body weight, there was no effect of photoperiod on chow fed rats, whereas the HFD fed rats were slightly but significantly less lean in SD than LD (p<0.001) and less lean than the chow fed rats (LD; p = 0.009, SD; p<0.001). The visceral organs showed similar responses with the liver and kidney both closely reflecting the decrease in absolute lean mass in SD and no effect of HFD (Fig. 2d,e).

### Blood hormone and metabolite levels

**Fig 3:**
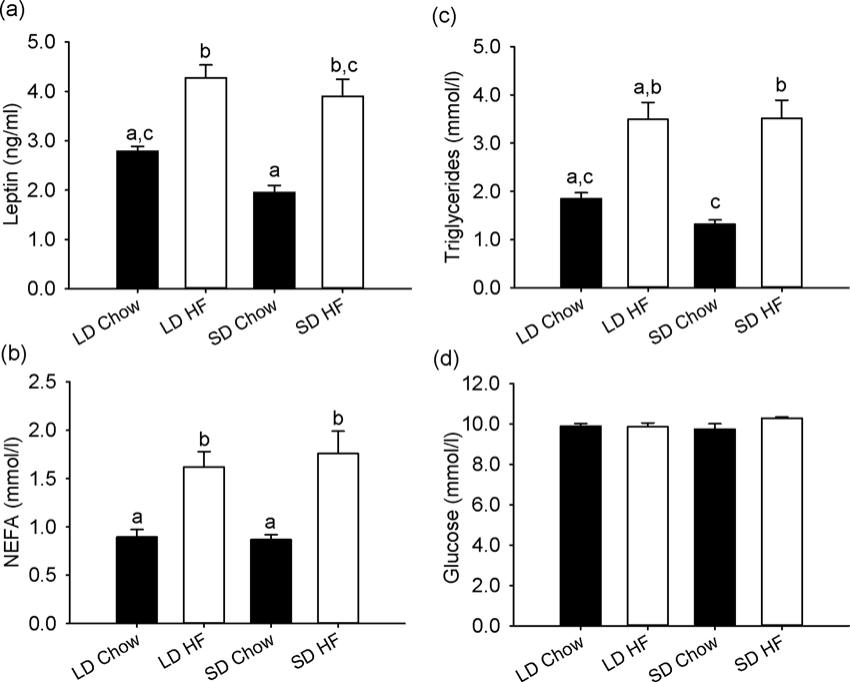
Effect of photoperiod and high fat diet (HFD) on hormone and metabolites levels in juvenile F344 rats after 4 weeks of treatment. (a-c): (a) serum Leptin, (b) plasma NEFA and (c) plasma Triglyceride levels were not changed by photoperiod but were significantly higher in HFD fed rats in both photoperiods (all p<0.05). (d) Glucose levels were unchanged with diet or photoperiod.

Serum levels of leptin, plasma levels of NEFA and TG were each unaffected by photoperiod in rats fed a chow diet; however each were markedly increased in rats fed HFD. Photoperiod had no additional effect (Fig. 3a-c). Blood levels of glucose were unaffected by diet or photoperiod (Fig. 3d).

### Hypothalamic gene expression

**Fig 4:**
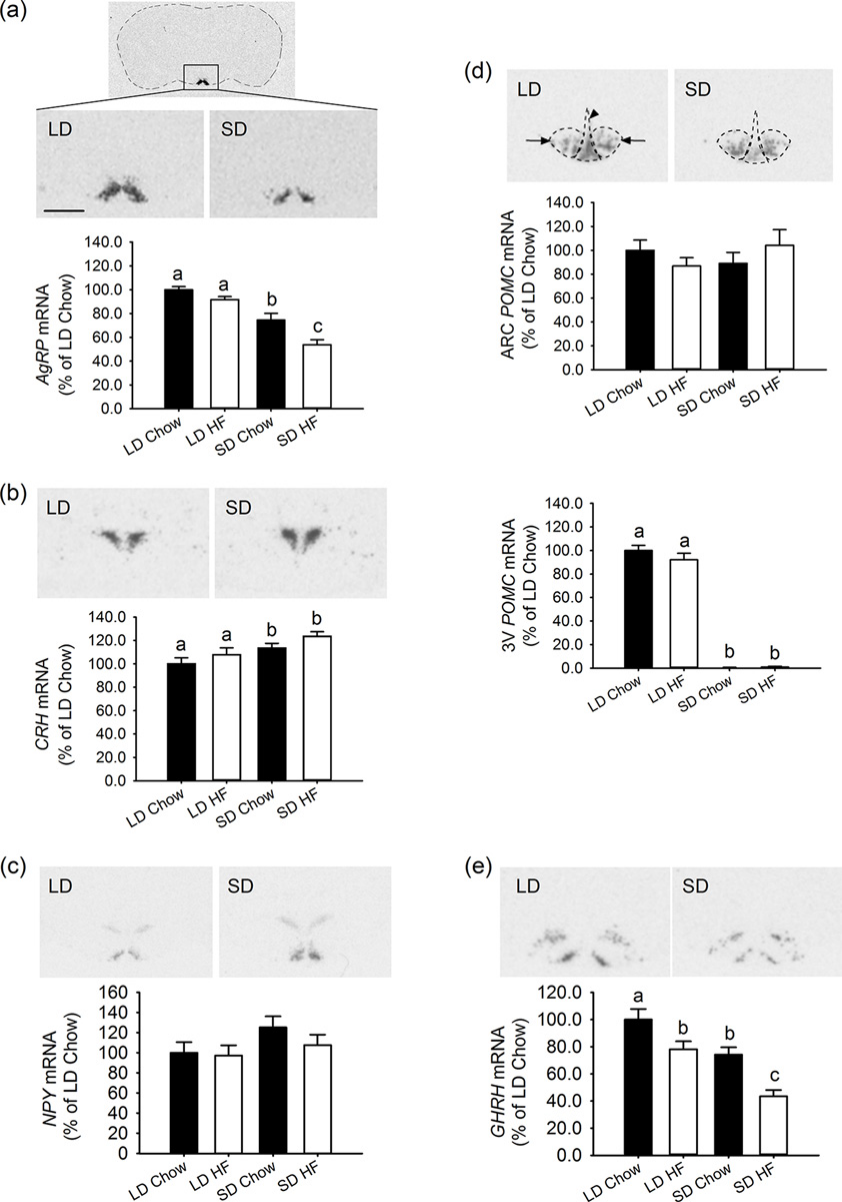
Effect of photoperiod and high fat diet (HFD) on hypothalamic expression of genes involved in energy balance growth in juvenile F344 rats 4 weeks of treatment, measured by quantitative in situ hybridisation. (a) *AgRP* mRNA expression quantified in the ARC was lower in SD than LD chow fed rats (p<0.001). The HFD had no effect in LD but reduced the *AgRP* levels further than chow fed rats in SD (p = 0.001). (b) *CRH* mRNA expression in the PVN was higher in SD than LD chow (p = 0.044) and HFD rats (p = 0.022). The HFD did not impact further on the expression levels in either photoperiod. (c) *NPY* mRNA levels tended to increase in SD chow fed rats in the ARC but the level did not reach significance (p = 0.119). There were no effects of HFD on *NPY* expression in either photoperiod. (d) *POMC* mRNA expression in the ARC is outlined and marked by arrows while atypical *POMC* expression in the ependymal layer of the 3^rd^ ventricle is marked by an arrowhead. In the ARC, *POMC* levels were unaffected by photoperiod or diet. In contrast, levels of *POMC* mRNA were markedly reduced in SD photoperiod (p<0.001) with no additional effects of HFD feeding. (e) *GHRH* mRNA expression in the ARC was lower in SD than LD chow fed rats. HFD reduced the *GHRH* expression in LD and even further in SD. Scale bar = 1.0mm for all images.

Photoperiod had a strong effect on the expression of genes involved in energy balance and growth within the hypothalamus after 4 weeks of treatment. Expression of the orexigenic gene, *AgRP*, in the ARC was lower in SD than LD (p<0.001), (Fig. 4a). Also expression of *CRH* (an inhibitor of food intake) in the PVN was increased in rats on SD relative to LD (p = 0.044), (Fig. 4b). For *AgRP*, HFD reduced the expression level further in SD (p = 0.001), (Fig. 4a), but no additional effect on *CRH* expression was observed with HFD feeding (Fig. 4b). These gene expression changes are consistent with a lower level of food intake in SD rats.

By contrast expression of the orexigenic gene *NPY*, in the ARC, tended to increase in SD relative to LD rats but the level did not reach significance in this study, whereas a significant increase in SD has been observed in our previous studies [7] (Fig. 4c). HFD had no effect on *NPY* gene expression. As previously described there are two distinct areas of expression for the *POMC* gene in the hypothalamus. The first is in the ARC, a region normally associated with its anorexigenic activity. Here there was no effect of either photoperiod or HFD on *POMC* gene expression (Fig. 4d). The second was in the ventral ependymal region, where strong suppression of *POMC* mRNA was observed in response to SD relative to LD (p<0.001), (Fig. 4d). This finding is concordant with a previous study [7]. However there was no effect of the HFD under either photoperiod (Fig. 4d).

The expression of *GHRH* in the ARC was affected by both photoperiod and HFD (Fig. 4e). Under SD there was lower expression of *GHRH* relative to LD (p = 0.007) with chow. The HFD further suppressed gene expression under both LD (p = 0.014) and SD (p = 0.002), (Fig. 4e).

### Growth hormone and IGF-1

**Fig 5:**
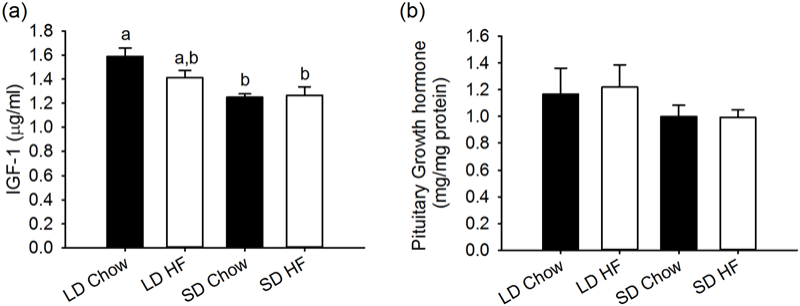
Effect of photoperiod and high fat diet (HFD) on serum IGF-1 and pituitary growth hormone (GH) levels in juvenile F344 rats after, 4 weeks of treatment. (a) IGF-1 levels in serum were lower in SD chow and HFD fed rats than LD chow fed while LD HFD fed rats were not significantly different. (b) Growth hormone levels in the pituitary were unaffected by either photoperiod or HFD.

Serum IGF-1 levels were 21.4% lower in F344 rats fed chow diets and exposed to SD for 4 weeks relative to LD treated animals (p<0.05). There was no difference in this response for animals given the HFD (Fig. 5a).

At a pituitary level, GH levels were unaffected by either photoperiod or diet (Fig. 5b).

### Photoperiodic signalling genes in the pars tuberalis and ependymal cells

**Fig 6:**
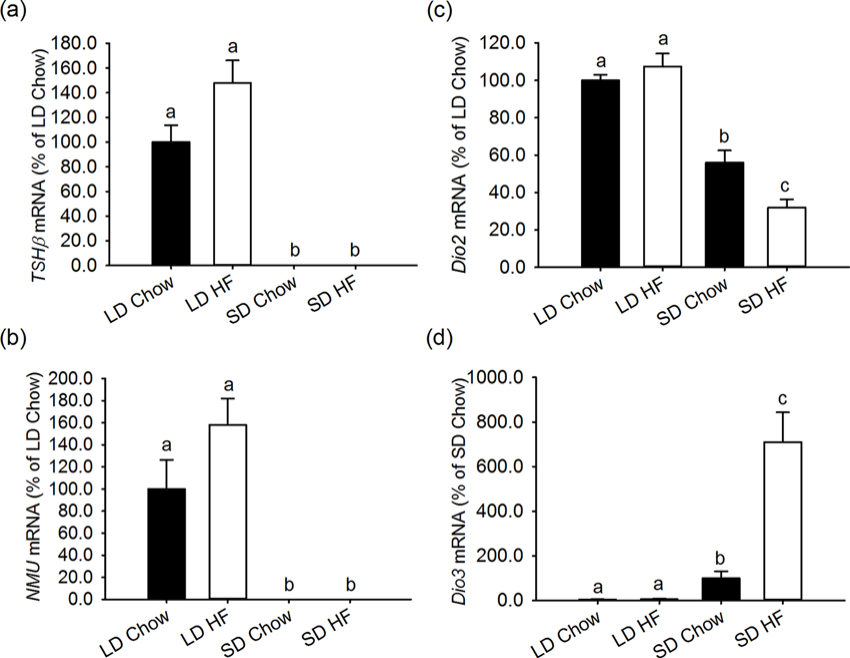
Effect of photoperiod and high fat diet (HFD) on gene expression of *TSHβ* and *NMU* in the pars tuberalis of the pituitary and Dio2 and Dio3 in the tanycytes around the third ventricle in juvenile F344 rats after 4 weeks of treatment. Gene expression was measured by quantitative in situ hybridisation. (a,b) Expression of *TSHβ* (a) and *NMU* (b) mRNAs in the pars tuberalis were significantly lower in both chow and HFD fed SD compared to LD rats. (c) *Dio2* mRNA levels in the ependymal cells around the 3^rd^ ventricle were lower in SD than LD chow and HFD fed rats (p<0.001). HFD reduced Dio2 further in SD compared to LD conditions (p<0.001). (d) *Dio3* mRNA expression was higher in SD than LD rats. HFD increased the *Dio3* mRNA level significantly in SD (p<0.001), but not in LD.

Using animals obtained in a separate study, which showed similar changes in body composition, the impact of photoperiod and HFD were studied on the expression of *NMU* and *TSHβ* in the pars tuberalis, as well as *Dio2* and *Dio3* expression in the ependymal cells around the third ventricle. As reported previously [36], both *TSHβ* and *NMU* are strongly expressed in animals on LD, but only weakly (undetectably) expressed in rats on SD. The HFD had no additional effect on these responses (Fig. 6a,b).

Within the hypothalamus, expression of *Dio2* and *Dio3* were found to be sensitive to photoperiod, with *Dio2* expressed more weakly and *Dio3* more strongly in SD relative to LD (both p<0.001) (Fig. 6c,d). HFD had no effect on the expression of either gene under LD conditions, yet strong effects under SD conditions. HFD caused a marked suppression of *Dio2* (p<0.001) (Fig. 6c) and a 7-fold increase in *Dio3* expression under SD (p = 0.001) (Fig. 6d).

## Discussion

This study reveals that photoperiodically sensitive and growing F344 rats are susceptible to mild obesity after being fed a HFD for 4 weeks with adiposity increasing from around 3–4% to 10% measured by MRI. This doubling of adiposity was also confirmed by the levels of fat associated with the epididymis, as well as by increased levels of serum leptin, TG and NEFAs. By contrast lean mass, measured as fat-free mass as well as organ weights for liver and kidney were affected only by photoperiod, with no effect of high fat diet over the same period. Whereas photoperiodic control of lean mass remained after feeding a HFD, photoperiodic regulation of fat mass was lost and these contrasting effects of HFD on the photoperiodic regulation of lean mass and adipose tissue accretion suggest that a primary effect of photoperiod in juvenile F344 rats is to set the trajectory for growth in these animals.

The effect of photoperiod, but not HFD, on serum IGF-1 levels, is consistent with this effect on lean mass. When fed a HFD, F344 rats adjust food and protein intake downward, as also observed by Togo et al. [25]. This suggests that F344 rats attempt to balance their food intake in terms of energy with the macronutrient (protein) requirements for growth. The resulting adiposity observed in this study, implies that while food intake is suppressed, when on a high fat diet, it is not possible to suppress intake sufficiently to prevent gain in body fat. The fact that rats suppress protein intake on a HFD relative to chow fed rats on the same photoperiod, yet manage to achieve the same lean mass, indicates that the HFD must modulate protein uptake or metabolism. Evidence from the literature suggests that a HFD alters not only the structure of the gut, but also can increase amino acid transport across the gut [37,38]. Thus it is likely that a HFD increases the efficiency of amino acid uptake and this could account for the lower level of protein intake on a HFD relative to chow fed rats.

These data contrast with a recent study, which reported F344 rats to be seemingly resistant to diet induced obesity (DIO), on the basis of measurements of epididymal fat and serum leptin levels, when fed a diet containing 55% fat. [25]. At present there is no obvious explanation for this difference in response of F344 rats to high fat diets between the two studies. Possible explanations may lie in differences in the high fat diets used and/or differences in the strain of F344 rat. In support of the latter we have previously reported differential responses to photoperiod in different strains of F344 rats [7], which indicates that F344 rats obtained from commercial suppliers/geographical locations are not genetically homogeneous.

The current study also revealed a clear interaction between HFD and photoperiod at the level of *GHRH* gene expression. In common with Zucker rats there is a reduction in hypothalamic *GHRH* expression, when fed a HFD [13,14]. Such an effect has not been seen in either mice or rats fed a HFD [17,15,16]. In those cases, *GHRH* gene expression was measured by Northern blotting or qPCR rather than in situ hybridisation used in this study. The latter is likely to provide more accurate assessment of gene expression in specific regions of the brain. Despite the lower *GHRH* expression in F344 rats on HFD under both LD and SD, there were no concomitant changes in serum IGF-1 levels over the 4 weeks of the experiment. Nonetheless IGF-1 was clearly suppressed by SD relative to LD, as previously described [7]. Since studies in mice have shown that it can take up to 8 weeks for effects of HFD on circulating IGF-1 levels to become apparent, with no observable change after 4 weeks of HFD [11], it is possible that changes in IGF-1 in F344 rats may become evident following a longer period of HFD feeding. It is also possible that the temporal mRNA expression profile for GHRH does not correlate fully with the temporal protein expression of GHRH, which could help to explain the lack of correlation of the serum IGF-1 and tissue GHRH mRNA responses.

Pituitary GH is a rather crude measure and despite clear effects of photoperiod on growth and serum IGF-1, there were no observable effects of photoperiod or HFD on pituitary GH. Since pulsatile GH is suppressed by HFD in other rodent models, even after only 4 weeks of treatment, and in humans [11], measurement of pulsatile GH is required to gain a proper assessment of the effects of photoperiod and HFD on GH status. Thus while the relative levels of serum IGF-1 correlate with the levels of fat free mass under the different conditions of photoperiod and HFD feeding, this study has shown that central inhibition of GHRH may be an early event in the pathogenic effects of a HFD on the somatotrophic axis.

A number of blood metabolites have been implicated in the regulation of the somatotrophic axis and these include glucose, leptin and NEFA [10]. The role of glucose in the regulation of the GH axis is contentious [10,8]. There is some evidence that glucose may suppress hypothalamic GHRH in diabetic rats [39], but the lack of change in glucose levels between chow fed and HFD rats in this study suggests that glucose does not play a major role in regulating GHRH. Leptin is a circulating hormone that increases with adiposity resulting from a HFD [40], but in humans serum GH levels are inversely correlated with leptin [41,42]. In rats, leptin is associated with activation of the GH axis [8,10,43], yet such a role is at odds with the results reported in this study. Plasma NEFA levels also increased with adiposity and they have been shown to inhibit the GH axis, by inhibiting GH secretion from the pituitary [44]. In this study while circulating NEFAs increased in rats on a HFD there were no associated changes in serum IGF-1 levels suggesting that there is no effect at the level of the pituitary or below. There is some evidence that NEFAs may influence the GH axis at the level of the hypothalamus [45] and thus the increased levels of NEFA may be causal in the suppression of hypothalamic GHRH, although this remains to be established [10].

Despite strong effects of HFD on food and protein intakes, other than for *AgRP* the expression of hypothalamic energy balance genes (*CRH*, *POMC* and NPY) were unaffected by HFD. While it is known that HFD can alter the circadian feeding behaviour of rats and mice there is no evidence that tissue rhythms in the hypothalamic SCN and arcuate nucleus are affected by HFD [46,47]. On this basis it is unlikely that effects of HFD may have been missed due to a phase shift in expression.

In contrast to the effects of HFD, photoperiod had strong effects on hypothalamic gene expression. *AgRP* gene expression in the ARC decreased in response to SD, consistent with a lower level of food intake [7] and the changes in *CRH* in the PVN are concordant with this response. CRH is known to inhibit food intake [48] and thus the increase in *CRH* gene expression under SD coupled with lowered *AgRP* gene expression are both consistent with a lower food intake drive under SD. In contrast, neither *NPY* nor POMC gene expression in the ARC were affected by photoperiod, suggesting that expression of these two ARC genes may be unrelated to changes in food intake on SD [7]. Nevertheless as previously reported atypical expression of the *POMC* gene in the ependymal layer is strongly affected by photoperiod, although its role in food intake and energy balance regulation is unknown [7]. Given that each of these genes can be strongly regulated by leptin [49], it is surprising that the HFD, and resulting obesity, had no effect on the patterns of gene expression of *NPY*, *POMC* or *CRH* under either photoperiod. It seems unlikely that this apparent lack of responsiveness to leptin is the result of insensitivity driven by the development of obesity. This is because the suppression in food intake in response to a HFD runs parallel to the food intake of the chow fed animals across the entire 4-week experimental period. If rats were becoming more leptin insensitive as a result of increased adiposity, then the suppression of food intake would be expected to have reduced with time on the HFD diet. This was not observed. Our understanding of the interaction between leptin and hypothalamic energy balance genes has been gained primarily from studies under conditions of food deprivation or from genetic models of obesity [50,51]. The results of this study indicate that the accepted feedback of leptin onto hypothalamic orexigenic /anorexigenic pathways [50,51], does not function as predicted under these circumstances. This raises important questions about how leptin and hypothalamic energy balance genes function in a physiological setting rather than in energy deficit mode or in mutant mice with genetic knock-outs of hypothalamic energy balance genes or leptin.

The effects of photoperiod on neuroendocrine function involve TSHβ and Dio2/3 in the pars tuberalis (PT) and tanycytes respectively [52,53,54,5]. It may also involve NMU, which is expressed in the PT and is under photoperiodic control [36]. The effects of photoperiod on *TSHβ*, *NMU*, *Dio2* and Dio3 gene expression are consistent with previous observations, namely LD increases *TSHβ*, *NMU* and *Dio2* expression and decreases *Dio3* expression relative to SD [36]. This would give rise to a net increase in hypothalamic T3 production under LD relative to SD. How this affects the neuroendocrine regulation of energy balance and growth is still unknown. While the HFD had no effect on hypothalamic energy balance genes, it did suppress *GHRH* gene expression under both photoperiods. It seems unlikely that these effects of a HFD on *GHRH* are mediated through changes in *Dio2* and *Dio3* gene expression or associated changes in *TSHβ* and *NMU*, as the pattern of gene expression changes in response to a HFD do not suggest a direct or simple correlative relationship. Instead it is more likely the effects of a HFD on *GHRH* involve mechanisms that are independent of the neuroendocrine pathways regulated by photoperiod.

Overall the results from this study clearly support the conclusion that over the short term, photoperiod exerts tight control of growth in F344 rats, in terms of lean mass accretion. To achieve the target rate of lean mass accretion (growth) set by photoperiod, it is implied that food intake is adjusted to match the protein requirements for growth. On a HFD, rats reduce their food intake, which suggests that protein uptake and assimilation must be more efficient on a HFD than on a chow diet. Nonetheless energy intake is increased leading to greater deposition of adipose tissue. This rebalancing of food intake, energy intake and protein intake, without overt effects on genes involved in the hypothalamic energy balance circuits, indicates that factors other than leptin must be involved in determining this new equilibrium when animals are placed on a HFD. By contrast a HFD exerts a suppressive effect on the growth axis at the level of the hypothalamus (GHRH), and it appears that these effects are independent of the photoperiod regulatory mechanisms. The effects on GHRH appear to be an early effect of a HFD on the somatotrophic axis and it seems likely that overt effects on serum IGF-1 levels and lean mass accretion will only be seen after prolonged exposure (>4 weeks) to a HFD.
